# Evaluation of pancreatic tumor development in *KPC* mice using multi-parametric MRI

**DOI:** 10.1186/s40644-018-0172-6

**Published:** 2018-11-08

**Authors:** Ravneet Vohra, Joshua Park, Yak-Nam Wang, Kayla Gravelle, Stella Whang, Joo-Ha Hwang, Donghoon Lee

**Affiliations:** 1 Department of Radiology, University of Washington, Seattle, USA; 2 Applied Physics Laboratory, University of Washington, Seattle, USA; 3 Department of Medicine, University of Washington, Seattle, USA

**Keywords:** Pancreatic ductal adenocarcinoma, Tumor microenvironment, *KPC*, Multi-parametric MRI

## Abstract

**Background:**

Pancreatic ductal adenocarcinoma (PDA) is a fatal disease with very poor prognosis. Development of sensitive and noninvasive methods to monitor tumor progression in PDA is a critical and unmet need. Magnetic resonance imaging (MRI) can noninvasively provide information regarding underlying pathophysiological processes such as necrosis, inflammatory changes and fibrotic tissue deposition.

**Methods:**

A genetically engineered *KPC* mouse model that recapitulates human PDA was used to characterize disease progression. MR measures of T_1_ and T_2_ relaxation times, magnetization transfer ratio (MTR), diffusion and chemical exchange saturation transfer were compared in two separate phases i.e. slow and rapid growth phase of tumor. Fibrotic tissue accumulation was assessed histologically using Masson’s trichrome staining. Pearson correlation coefficient (r) was computed to assess the relationship between the fibrotic tissue accumulation and different MR parameters.

**Results:**

There was a negative correlation between amide proton transfer signal intensity and tumor volume (*r* = − 0.63, *p* = 0.003) in the slow growth phase of the tumor development. In the terminal stage of rapid growth phase of the tumor development MTR was strongly correlated with tumor volume (*r* = 0.62, *p* = 0.008). Finally, MTR was significantly correlated with % fibrosis (*r* = 0.87; *p* < 0.01), followed by moderate correlation between tumor volume (*r* = 0.42); T_1_ (*r* = − 0.61), T_2_ (r = − 0.61) and accumulation of fibrotic tissue.

**Conclusions:**

Here we demonstrated, using multi-parametric MRI (mp-MRI), that MRI parameters changed with tumor progression in a mouse model of PDA. Use of mp-MRI may have the potential to monitor the dynamic changes of tumor microenvironment with increase in tumor size in the transgenic *KPC* mouse model of pancreatic tumor.

**Electronic supplementary material:**

The online version of this article (10.1186/s40644-018-0172-6) contains supplementary material, which is available to authorized users.

## Background

Pancreatic ductal adenocarcinoma (PDA) is the most lethal form of human cancer [1]. Desmoplasia, a hallmark pathologic feature of PDA, is characterized by the presence of a robust stroma containing fibroblasts and inflammatory cells [2]. Diagnosis of pancreatic cancer is usually made at later stages of the disease making it even more difficult to treat. Clear understanding of tumor progression may help in identifying PDA at an early stage. Therefore, in order to characterize the tumor progression, we need 1) to investigate tumor development in an effective preclinical model that closely mimics human disease progression and 2) a sensitive, non-invasive monitoring tool that would provide detailed information regarding the disease progression and can be used in future clinical studies.

As an experimental model for pancreatic cancer, tumors have been implanted subcutaneously and within the pancreas [3–5]. However, both of these models do not parallel the human disease progression. A genetically engineered mouse model, having genetic alterations in genes K-ras^LSL-G12D/+^; Trp53^LSL-R172H/+^; Cre (KPC) offers an alternative to transplantation models for preclinical therapeutic evaluation as it expresses mutations similar to human pancreatic cells [6] and develops pancreatic tumors in which pathophysiology and molecular features resemble those of human PDA [7]. There is a dire need to develop non-invasive techniques to monitor disease progression as well as study the therapeutic effects of existing and novel chemotherapeutic agents.

Magnetic resonance imaging (MRI) has been proven to be extremely useful in clinical trials for monitoring tumor development [8] and assessing therapeutic effects of novel therapeutic strategies [9–11]. Currently, management decisions in almost all phases of diagnosis, treatment, and follow-up rely on gold standard magnetic resonance (MR) measures i.e. spin-lattice relaxation time (T_1_) and spin-spin relaxation time (T_2_) [12, 13]. However, T_1_ and T_2_ measures alone may not be sensitive enough to investigate the entire spectrum of tumor properties. Recently, diffusion weighted imaging (DWI) [14, 15] and magnetization transfer imaging (MTI) [16–18] have been used as complementary imaging techniques to conventional MR measures to characterize adenocarcinoma. Finally, amide proton transfer (APT) imaging based on the chemical exchange saturation transfer (CEST) approach, which provides valuable information regarding the tumor microenvironment, has drawn considerable attention as a novel MRI contrast agent in the field of molecular imaging [19, 20]. To monitor the dynamic microenvironment of the tumor as in PDA, we need to employ mp-MRI to understand the disease progression, and the tumor environment thereby helping to plan the appropriate therapeutic regimen. In fact, several studies have demonstrated that multiparameteric MR based approach enables better quantitation of pathological processes in abdominal solid organs [21–23].

Based on the promising results of these studies, we hypothesized the mp-MRI may enable to accurately quantify the disease progression in *KPC* mice. In this study, we conducted MRI at a high field strength of 14 Tesla (T) utilizing the T_1_, T_2_, DWI, MTI and CEST imaging sequences to monitor the progression of pancreatic ductal adenocarcinoma in *KPC* mice. Additionally, we compared MR parameters with the histologic markers of fibrotic tissue accumulation in pancreatic tumors in the *KPC* mouse model.

## Methods

### Animal handling and care

The study was conducted with the approval from our institutional animal care and use committee (IACUC). *KPC* mice (*n* = 16) were enrolled in the study when they had a small palpable mass, which was confirmed by ultrasound imaging and MR imaging. All mice were imaged upon enrollment and at the final time point when the tumor reached terminal size (10 mm in any one direction). A subgroup of *KPC* mice (*n* = 9) were imaged weekly between the baseline and final time points. All mice were euthanized at the terminal time point and the tumor and associated pancreatic tissue was excised and prepared for histological evaluation.

### MR data acquisition

MRI experiments were performed on a 14 T Bruker Avance 600 MHz/89 mm wide-bore vertical MR spectrometer (Bruker Corp., Billerica, MA). A birdcage coil (inner diameter 25 mm) was used to image the animal mounted on a cradle with a respiratory monitoring probe. Animals were anesthetized before being secured to the custom-built cradle. The coil was then inserted vertically into the scanner. For the entire duration of the experiment core body temperature of mice was maintained at 37 °C. The entire MR data acquisition took 50–60 min during which the animals were continuously monitored for respiratory rate. All the images were gated to respiration of the animal. Following the MR acquisition, mice were removed from the coil and allowed to recover. Longitudinal MRI was implemented to measure pancreatic tumor growth in *KPC* mice (*n* = 9) over a period of 6 weeks. Mice included in longitudinal MRI had a palpable mass at the baseline measurement, which was measured by ultrasound (US) and later corroborated using MRI.

#### Anatomical images

The multi-slice MRI protocol, covering the whole tumor, started with fat-suppressed T_1_ weighted coronal images (repetition time (TR) = 2000 ms, echo time (TE) = 5.49 ms, number of averages (NA) = 1, field of view (FOV) = 30 × 30 mm^2^, rare factor = 8, matrix size = 128 × 256; yielding spatial resolution of 0.234 × 0.117 mm/pixel) for anatomical reference. Subsequently, 20 axial images were acquired to calculate tumor volume.

#### T_1_ mapping

Multiple images using rapid acquisition with refocused echoes (RARE) were acquired using following parameters: TE = 9.66 ms, TR = 5500, 3000, 1500, 1000, 385.8 ms, NA = 1, FOV = 30 × 30 mm^2^, rare factor = 2, matrix size = 256 × 128 (reconstructed phase encoding steps = 128; acquisition phase encoding steps = 96) yielding spatial resolution of 0.117 × 0.234 mm/pixel. The data acquisition time was approximately 9 min.

#### T_2_ mapping

Multiple spin-echo data were acquired in coronal orientation covering the area from liver to kidneys. The quantitative T_2_ maps were generated using a multi-slice multi echo sequence, with fat signal suppressed, utilizing following parameters: TR = 4000 ms; TE = 12 echoes equally spaced from 6.28 ms to 75.4 ms; NA = 1; FOV = 30 × 30 mm^2^; matrix size = 256 × 128 (reconstructed phase encoding steps = 128; acquisition phase encoding steps = 91) yielding spatial resolution of 0.117 × 0.234 mm/pixel. To cover the entire abdominal region, 10 contiguous slices were acquired without any inter slice gap. The data acquisition time was approximately 6 min.

#### Magnetization transfer (MT)

MT ratios (MTR) were acquired using a gradient echo sequence (TR/TE = 625/2 ms, flip angle = 30°) with an off-resonance frequency of 7000 Hz and a saturation pulse block pulse shape, 50 ms width, and 10 μT amplitude. A series of 10 images were acquired with FOV = 30 × 30 mm^2^, matrix size = 256 × 256 yielding spatial resolution of 0.117 × 0.117 mm/pixel. The acquisition time for data acquisition was approximately 3 min.

#### Diffusion weighted imaging (DWI)

An echo planar imaging (EPI) diffusion measurement (echo train length = 16, pulse duration = 3.0 ms and diffusion time = 7.46 ms) was performed to acquire series of 10 slices using following parameters: TR = 2500 ms; TE = 17.7 ms; NA = 1; FOV = 30 × 30 mm^2^; matrix size = 128 × 128 yielding spatial resolution of 0.234 × 0.234 mm/pixel. Diffusion weighted measurements were acquired with 8 different b values (0, 30, 60, 100, 150, 200, 300, 500 s/mm^2^). The data acquisition time was 2 m 40s.

#### Chemical exchange transfer saturation (CEST) imaging

On a single 1 mm slice, delineating tumor, amide proton transfer (APT) imaging was performed with respiratory gating using small animal monitoring device (SA instruments, Inc., Stony Brook, NY). CEST imaging data were acquired using RARE sequence (continuous-wave block pulse, B1 = 0.5 μT, duration = 2 s), which was applied at 25 frequency offsets from − 360 Hz to 360 Hz with an interval of 0.5 ppm to estimate a center frequency shift (water saturation shift referencing (WASSR) approach) [24–26]. Other imaging parameters were: TR/TE = 2200/7 ms, FOV = 30 × 30 mm^2^, matrix size = 128 × 128, flip angle = 180^o^, and number of excitations = 1. For saturation a single slice was acquired with 6 frequency offsets at ±3.0, ±3.5, ±4.0 ppm, with an off-resonance RF pulse applied for 3 s at a power level of 2 μT. Other parameters were: TR/TE = 5000/7 ms, matrix = 128 × 128 (reconstructed phase encoding steps = 128; acquisition phase encoding steps = 96), FOV = 30 × 30 mm^2^, rare factor = 8. Finally, a control image with the saturation offset at 300 ppm was also acquired. Total acquisition time for each animal was approximately 19 min.

### Ultrasound (US) data acquisition

The animal was anaesthetized, placed in the supine position and images of the whole tumor were acquired (550 s probe, Vevo 2100, Fujifilm Visualsonics, Toronto, Canada). Ultrasound images were taken every 0.5 mm, in the transverse plane, throughout the whole tumor. The area of the tumor in each image was determined using Vevo LAB v2.1.0. The tumor volume was calculated by multiplying the area with the inter-slice distance.

### Image analyses

All raw MR images were processed using Image-J software (http://imagej.nih.gov/ij/), to measure mean values of the different tumors. Regions of interest (ROI) were drawn to circumscribe the entire tumor (Additional file 1: Figure S1). *Anatomical Images* were used to measure tumor volume. Tumor volume was measured and reconstructed using Amira (Visualization Sciences Group, Burlington, MA), a 3-D software platform [18]. *T*_**1**_ *and T*_**2**_ *maps*: Maps were generated using T_1_ and T_2_ weighted images. *MTR maps*: The MTR was measured using the following ratio: (SI_0_ - SI_s_/SI_0_), where SI_0_ represents the tissue signal intensity without saturation pulse applied while SI_s_ represents the tissue signal intensity with saturation pulse. *Diffusion maps*: Diffusion weighted MR signal decay was analyzed using mono-exponential model: S_b_/S_0_ = exp.(−b∙ADC). Where S_b_ is the MRI signal intensity with diffusion weighting b, S_0_ is the non-diffusion-weighted signal intensity and ADC is the apparent diffusion coefficient. In addition to mono-exponential model, a bi-exponential model was used to estimate intra voxel incoherent motion (IVIM) related parameters of perfusion fraction (or pseudo-diffusion) and diffusion [27]. Three lowest b values of 0, 30 and 60 s/mm^2^ were used to calculate perfusion component (or pseudo-diffusion) whereas rest of the 5 b values of 100, 150, 200, 300 and 500 s/mm^2^ were used to calculate tissue diffusivity component. Finally, *APT-MR* images were quantified using the following equation: [S_sat_ (− 3.5 ppm) – S_sat_ (3.5 ppm)]/S_0_ where S_sat_ and S_0_ are the water signal intensities measured with and without saturation pulse.

### Histogram analyses

Regions of interest were selected across consecutive three slices having greatest tumor area to increase the coverage and improve the reliability. For each tumor, values from each slice were combined to generate a single histogram. Histograms were then normalized by plotting the percentage of pixels remaining above the specific measurement in the x-axis, generating a cumulative histogram. Finally, the area under curve (AUC) with units of % pixels x parameter on x-axis was used to compare values in two different groups.

### Histological analysis

After extracting the tumor, it was immediately embedded in optimum cutting temperature (OCT) medium. Three serial, 8-μm thick sections were cut every 1 mm through the entire tumor (CM1950, Leica Biosystems Inc., Buffalo Grove, Illinois) and the sequential sections at each level were stained with Masson’s trichrome to visualize fibrotic tissue deposition. The three sections, matching with MR images, were used for fibrotic tissue quantification. All sections were examined using a Nikon 80i upright microscope (Nikon, Melville, New York). Images of whole sections stained with Masson’s trichrome were acquired with a 10 x objective lens. Fibrotic tissue, identified by blue staining, was separated by thresholding of the hue (130–190), saturation (20–255), and brightness (10–240) values using ImageJ (ImageJ 1.42 National Institutes of Health, Bethesda, MD). The fibrotic tissue content is presented as the percent area of fibrosis over the whole tumor area. High resolution, 40 μm thick, cryo-images of one of the tumor bearing mice were acquired at Bioinvision® (www.bioinvision.com).

### Statistical analysis

Statistical analysis was performed using Graph Pad prism version 6.0 (GraphPad, La Jolla, CA, USA). Tumor volume was compared between baseline and final time points using paired t test. Pearson correlation coefficient (r) with Bonferroni correction was computed to assess the relationship between the tumor volume and different MR parameters. All data were presented as means and standard deviations (SD). Statistical significance was accepted for *p* < 0.05.

## Results

### Increase in pancreatic tumor volume with age in *KPC* mice

**Fig. 1:**
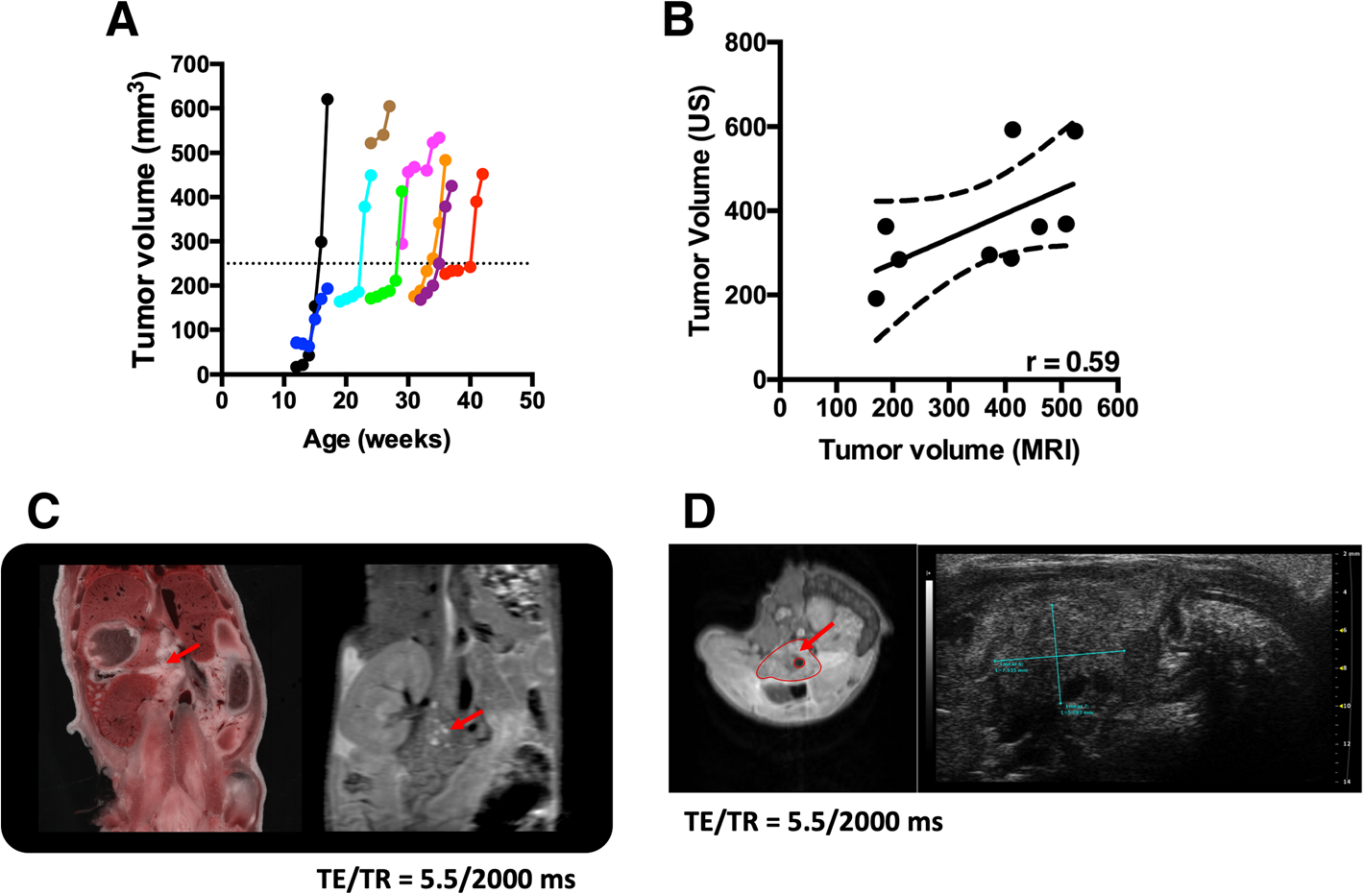
**a** Progressive tumor volumes for individual *KPC* mice (*n* = 9) that were imaged longitudinally. There was an exponential increase in tumor volume, in 6 out of 9 animals, once it reached a certain threshold value i.e. 250 mm^3^ (dotted line) in this study. **b** Correlation between US and MRI measurements for tumor volume. **c** Representative whole body image of tumor in *KPC* mice (Bioinvision, Cleveland, OH) and coronal MR image of the same mouse. **d** Representative axial MR and US image of tumor in the same *KPC* mouse. Red arrows point towards pancreatic tumor (**c** and **d**)

Longitudinal MRI demonstrated two distinct phases existed in tumor progression in *KPC* mice i.e. slow phase and rapid phase. Tumor growth, measured by MRI, in 6 out of 9 *KPC* mice increased exponentially once it surpassed a threshold value of 250 mm^3^ (Fig. 1a). Overall, there was a significant increase in tumor mass from baseline measurements (205.5 ± 154.4 mm^3^, mean ± SD) to the 6-week time point (455.9 ± 137.3 mm^3^). A moderate correlation was found between volume measurement by US and MRI (*r* = 0.59) (Fig. 1b). Finally, a high-resolution MR coronal section was compared with a whole body cryo-image (Fig. 1c) and MR axial section was compared with axial US image (Fig. 1d) to compare spatial resolution of different modalities.

### MR parameters correlate with tumor volume

**Fig. 2:**
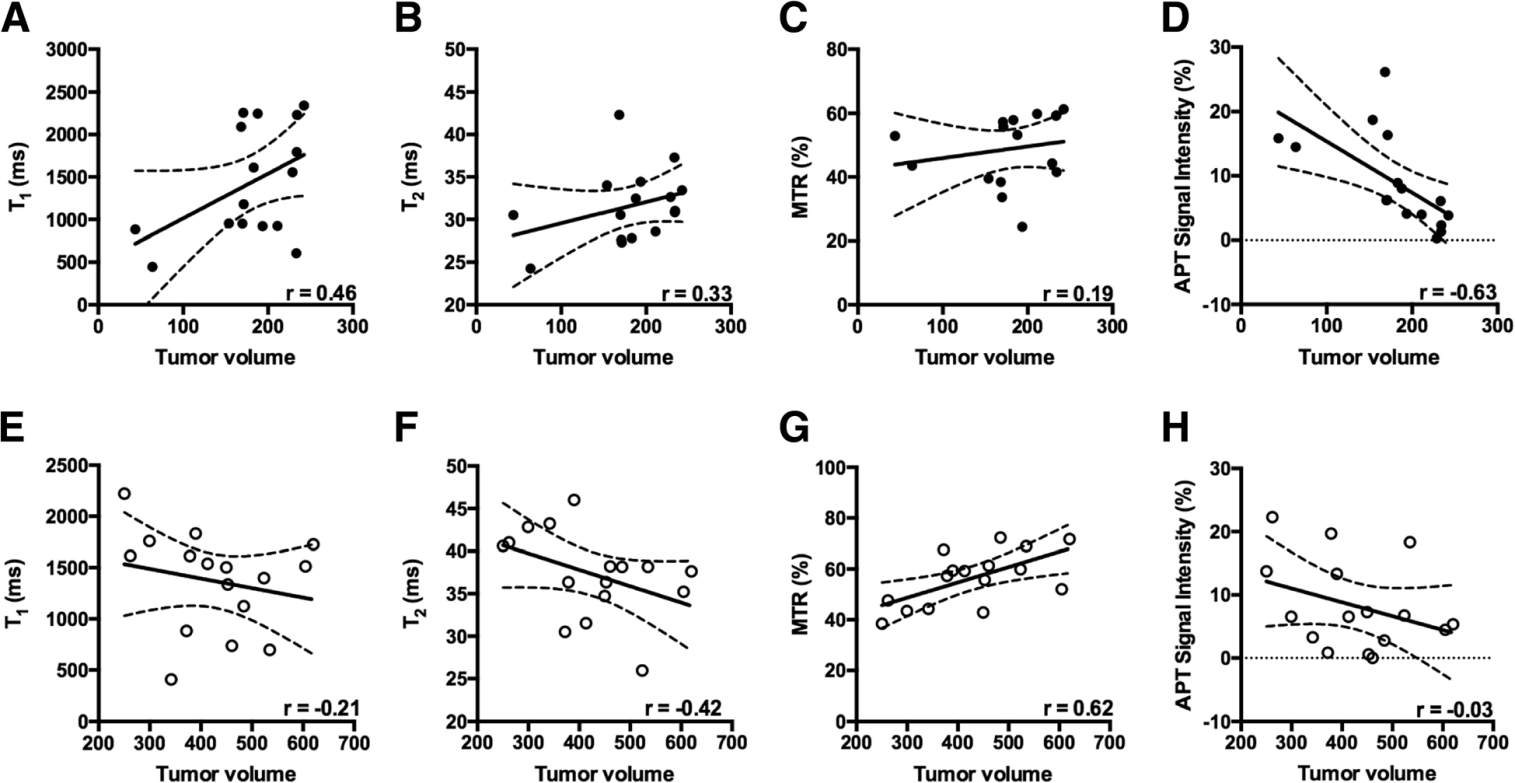
Relationship between tumor volume and different MR parameters. **a**-**d** show the relationship between tumor volume and T_1_, T_2_, MTR and APT signal intensity respectively, when the tumor volume is less than 250 mm^3^. **e**-**h** show the relationship between tumor volume and T_1_, T_2_, MTR and APT signal intensity respectively, when the tumor volume exceeds 250 mm^3^

**Fig. 3:**
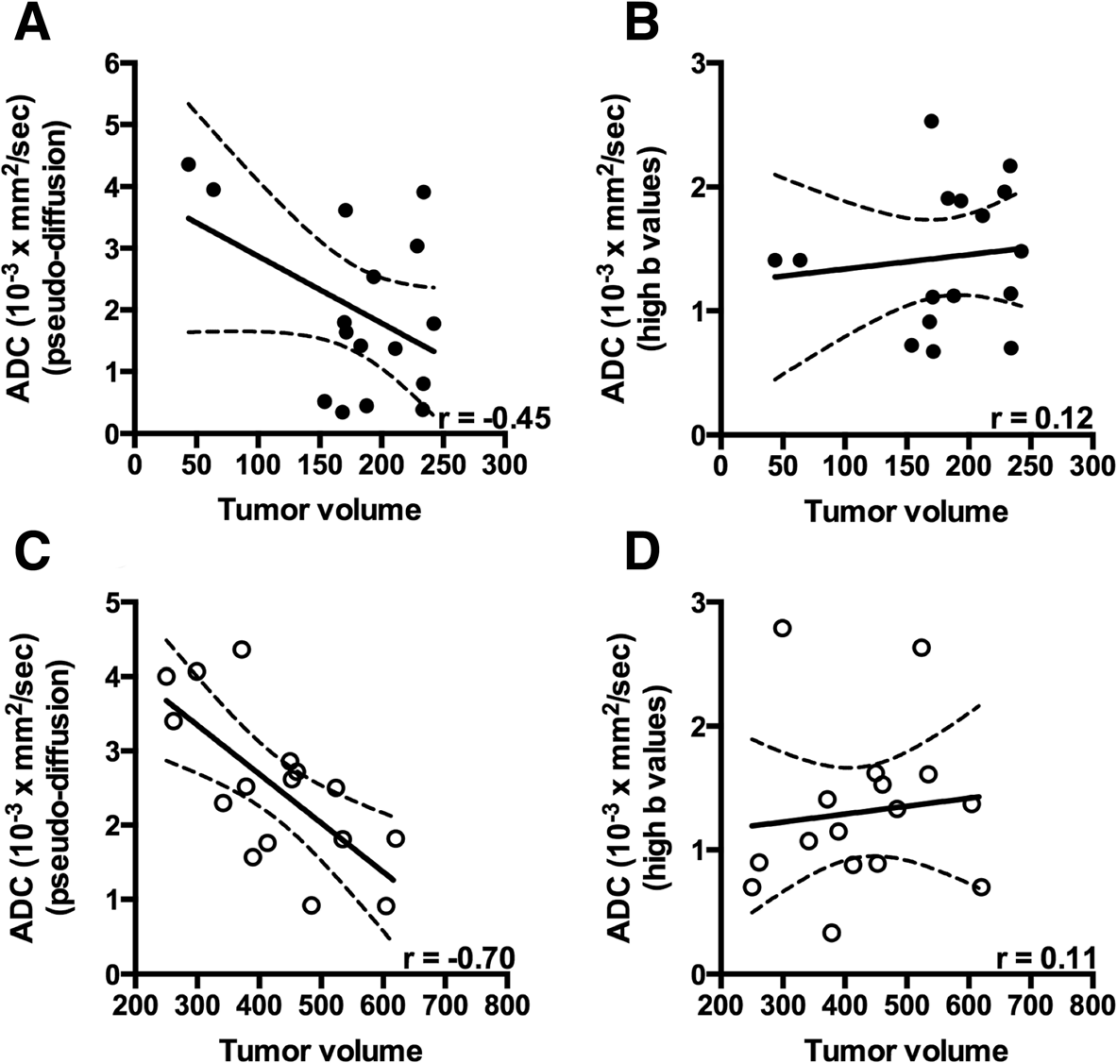
Relationship between tumor volume and diffusion measurements. **a** and **b** show the relationships between tumor volume and ADC (pseudo-diffusion), and ADC (high-b values), respectively, when the tumor volume is smaller than 250 mm^3^. **c** and **d** show the relationships between tumor volume and ADC (pseudo-diffusion), and ADC (high-b values), respectively, when the tumor volume is larger than 250 mm^3^

Multi-parametric data was analyzed based on the size of the tumor i.e. < 250 mm^3^ and > 250 mm^3^ as shown in Figs. 2 and 3. We chose a threshold value of 250 mm^3^ to differentiate between a slow and rapid growth phase in tumor development. Tumor size is an important prognostic factor in various cancers. Indeed, studies have documented a certain cut off size in various tumors enhance the prognosis [28–30]. Furthermore, there was a strong correlation between tumor volume and amide proton transfer (APT) signal intensity (*r* = − 0.63, *p* = 0.003) in < 250 mm^3^ whereas no correlation was present between APT signal intensity and tumor volume (*r* = − 0.03) when the tumor size was > 250 mm^3^. When the tumor size surpassed the threshold value i.e. 250 mm^3^, magnetization transfer ratio (MTR) displayed strong positive correlation with the tumor volume (*r* = 0.62, *p* = 0.008). Based on intravoxel incoherent motion (IVIM) model, [27, 31, 32] pseudo-diffusion or perfusion component (using low-b value) showed a strong negative correlation with the tumor volume (*r* = − 0.70, *p* = 0.003) whereas we did not find any correlation between diffusion (high-b values) and tumor volume (*r* = 0.12; < 250mm^3^, *r* = 0.11; > 250 mm^3^).

### Histogram analyses of tumor progression

**Fig. 4:**
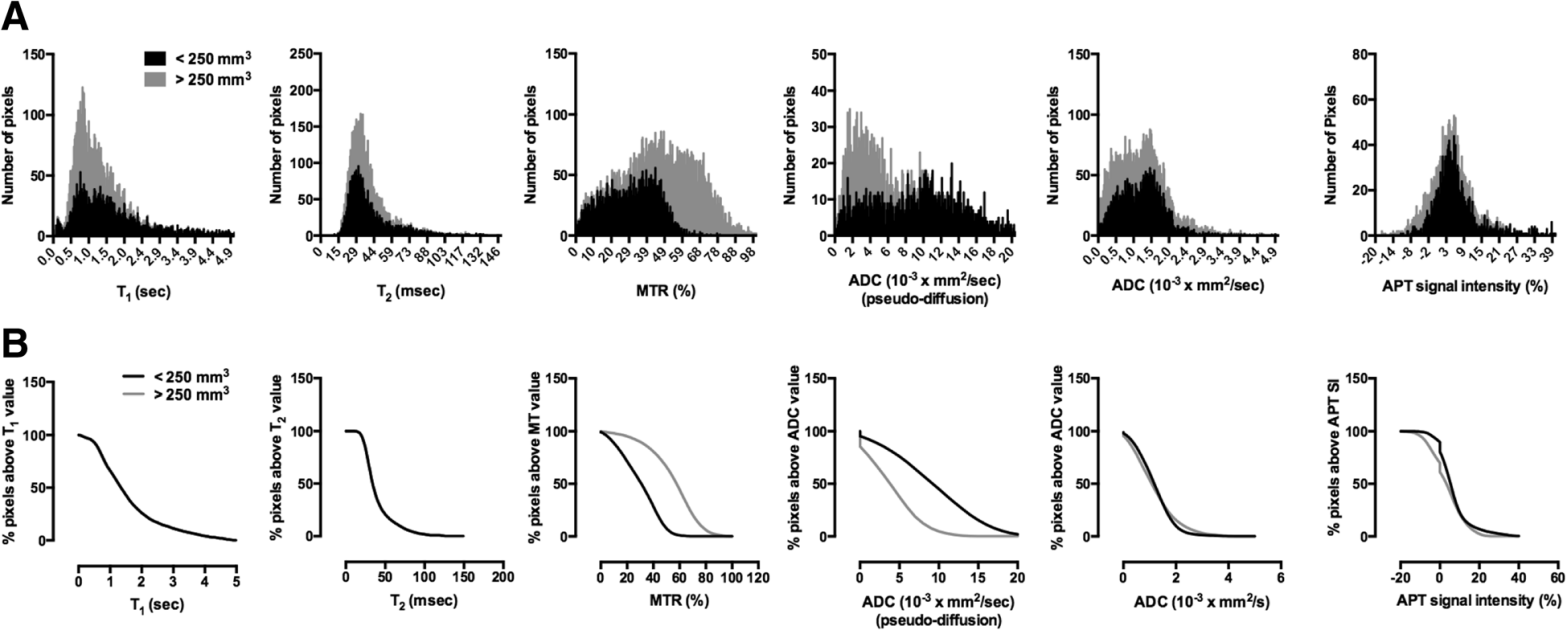
**a** Representative figure of frequency distribution of the pixels of different MR parameters in a *KPC* mouse at baseline and final time points. **b** Representative figure of percentage pixels above T_1_, T_2_, MTR, ADC and APT value in a KPC mouse at baseline and final time points

**Fig. 5:**
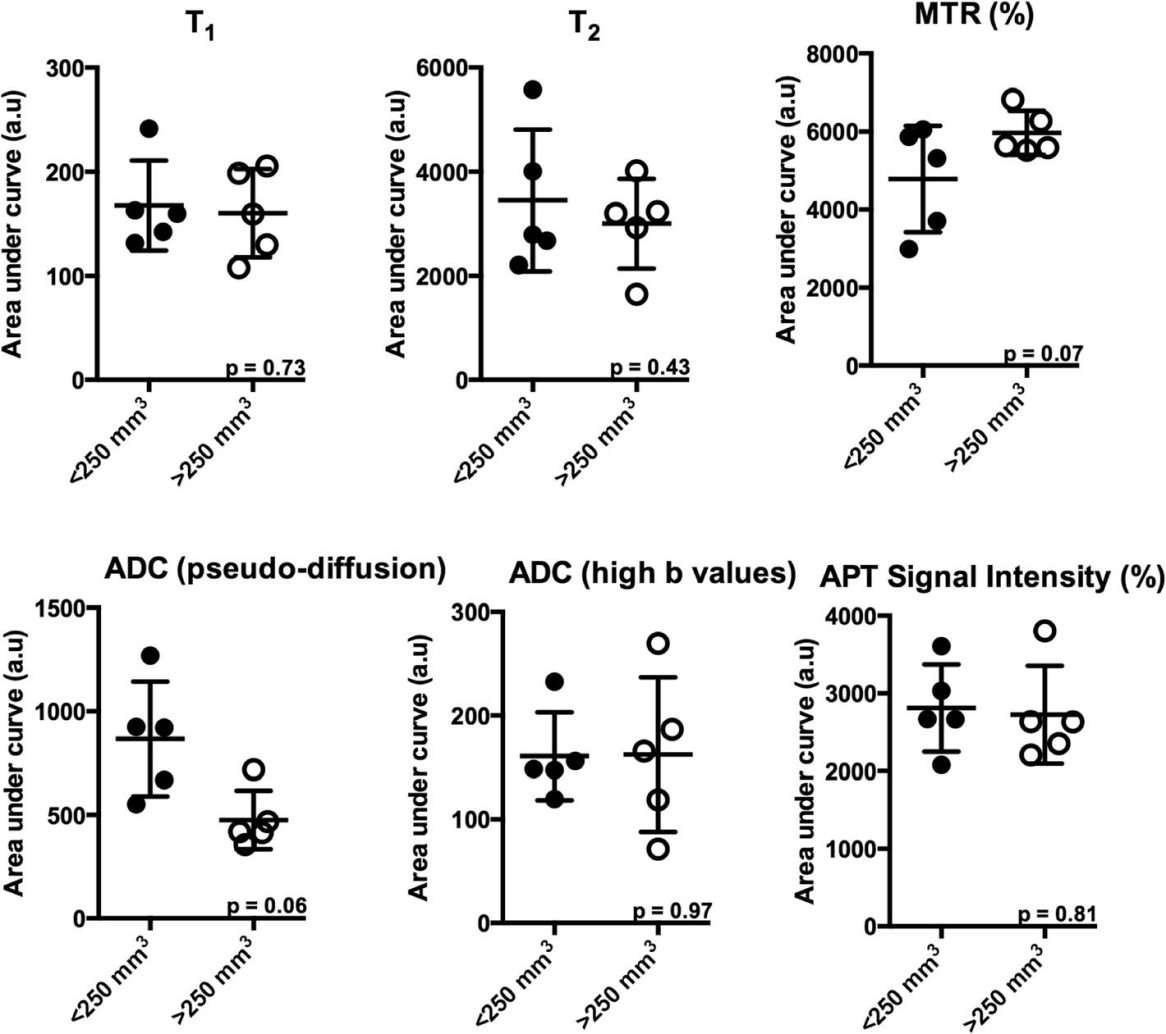
Area under curve (AUC) demonstrating differences in multi-parametric MR measures in PDA of < 250 mm^3^ and > 250 mm^3^ in 5 *KPC* mice

To assess pixel-pixel changes in MR parameters regionally, histogram analysis was implemented in the tumor area of *KPC* mice. Histograms were generated for T_1_, T_2_, MTR, and apparent diffusion coefficient (ADC) by combining values from 3 different slices for the pancreatic tumor. There was a general shift toward higher values for MTR and lower values for ADC in a larger tumor group i.e. > 250 mm^3^ (Fig. 4a). Additionally, the difference between smaller and larger tumor groups was better visualized in a cumulative histogram, which was produced by plotting the percentage of pixels remaining above the x-axis values (Fig. 4b). Finally, area under curve (AUC) was calculated from cumulative histograms for mp-MRI did not show any significant difference between < 250 mm^3^ and > 250 mm^3^ (Fig. 5).

### Histological analysis

**Fig. 6:**
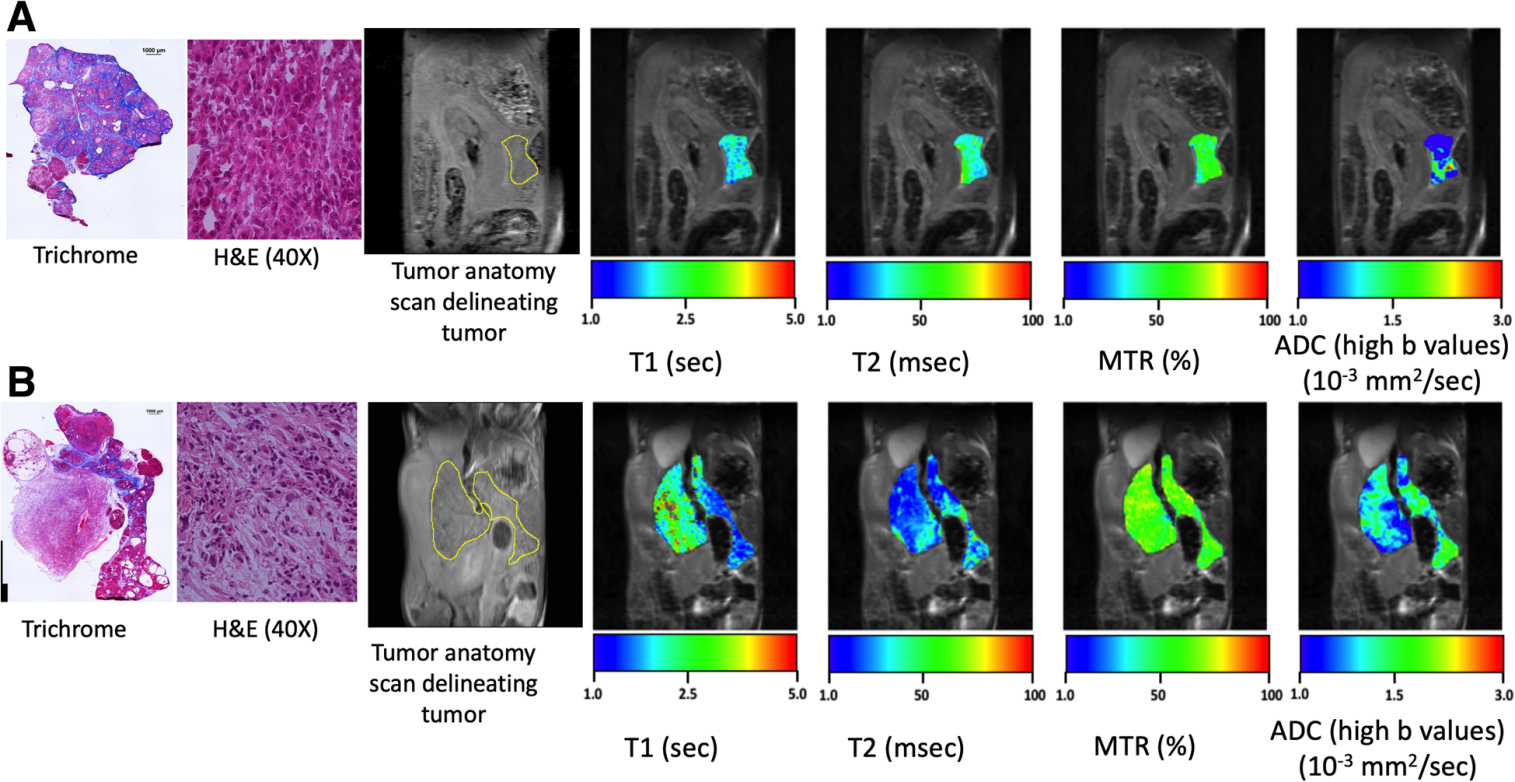
Representative Masson’s Trichrome and H&E stains for a smaller tumor (**a**) and larger tumor (**b**) from *KPC* mice and corresponding anatomic images and colored maps with T_1_, T_2_, MTR and ADC measures for pancreatic tumor

**Fig. 7:**
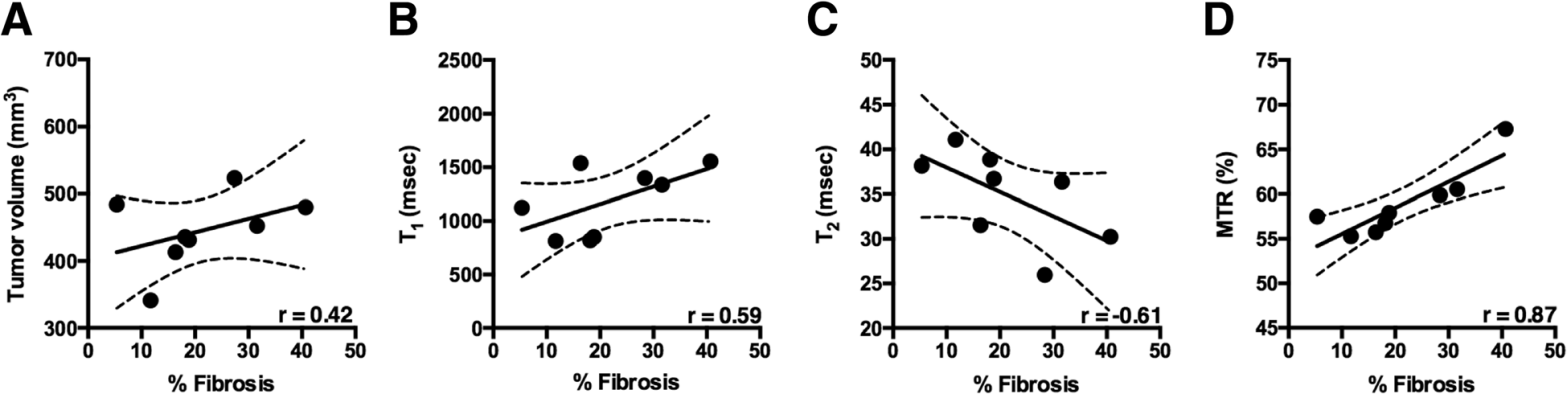
Correlation between % fibrosis and tumor volume and other MR parameters. There was moderate correlation between increase in tumor volume, T_1_, T_2_ and increase in fibrotic tissue accumulation (**a**, **b**, **c**). MTR % was significantly correlated with increase in fibrotic tissue accumulation (**d**)

Fibrotic tissue deposition was identified by Masson’s trichrome stained tumors and compared to mp-MR maps (Fig. 6). There was a significant correlation between MTR and % fibrosis (*r* = 0.87; *p* < 0.01), followed by moderate correlation between tumor volume (*r* = 0.42; *p* = 0.30), T_1_ (*r* = 0.59, *p* = 0.13), T_2_ (*r* = − 0.61, *p* = 0.10) and fibrotic tissue deposition (Fig. 7).

## Discussion

The present study evaluated multi-parametric MRI (mp-MRI) to monitor pancreatic tumor progression in the *KPC* mouse model. MR measurements consisted of T_1_, T_2_, ADC, MTR and APT imaging. The results from the present study revealed that 1) the tumor size increases exponentially once it crosses a threshold value i.e. 250 mm^3^; 2) MTR (%) and ADC (pseudo-diffusion) demonstrated higher correlation with tumor volume compared to other measures especially during the later stages of tumor development; and 3) MTR was significantly correlated with increase in fibrotic tissue accumulation.

We have demonstrated  a moderate correlation between US and MRI measures of tumor volume. Although US has been used extensively as an imaging tool in small animals, poor signal-to-noise ratio (SNR) images is one of the major limitations to ultrasound use. On the other hand, MRI has the ability to provide images in great anatomic detail. Decreased SNR could be one of the reasons that explains a moderate correlation between tumor volume measurements using US and MRI. Additionally, tumor burden or size has been demonstrated to be an important prognostic factor among patients with different types of carcinomas [28–30]. Furthermore, it has been suggested that tumors grow in a non-linear fashion [33]. Similarly, in the present study we have demonstrated that the growth of tumors follows a non-linear trend i.e. in two separate phases i.e. slow and rapid. Finally, we found that a cut off size of 250 mm^3^ in the *KPC* mouse model may provide enhanced prognosis.

The measurement of any MR parameter in isolation may undermine the dynamic nature of tumor microenvironment. However, the use of mp-MRI enables the evaluation of multiple parameters to give a more representative picture of the tumor microenvironment. Methods such as quantitative T_1_, T_2_, ADC and MTR are well established methods used to characterize tumor progression [8, 34–36]. T_1_ and T_2_ relaxation times of water molecules in tissue have been demonstrated as sensitive indicators of tumor progression as well as responses to different therapeutic agents [35, 37]. T_1_ is sensitive to factors such as the amount of 1) water in the extracellular space and 2) protein in the water [38]. Similarly, the results of our study demonstrate a moderate positive correlation between T_1_ and tumor volume in the smaller tumor group (< 250 mm^3^) suggesting increased water and protein content especially during the earlier stages of tumor development. Conversely, when the tumor size increased beyond the threshold value (250 mm^3^), T_1_ failed to provide detailed information regarding the tumor development. In advanced tumor stages there is more fibrotic tissue deposition when compared to earlier stages [39], which can be demonstrated by a decrease in T_2_ relaxation time. Similarly, in the present study, we revealed a moderate negative correlation between T_2_ and tumor volume in the larger tumor group and a decrease in T_2_ relaxation time was also correlated to an increase in fibrotic tissue content. These findings suggested that fibrotic tissue accumulated during later stages of disease progression and that T_2_ is more sensitive than T_1_ to tumor progression, especially in terms of fibrotic tissue accumulation.

DWI is sensitive to the thermally driven motion of water molecules along the orientation of additional diffusion sensitizing gradients applied during MR sequence. The signal attenuation coefficient, known as apparent diffusion coefficient (ADC) can be derived from diffusion weighted images. ADC has been shown to be sensitive to tissue structure at the cellular level [40, 41]. Results from the present study revealed only moderate correlation between tumor volume and ADC in larger tumor group suggesting that tumor environment becomes more restrictive as tumor size increases. Moreover, a study by Muraoka et al. demonstrated significant differences in ADC between areas of sparse and dense fibrotic area [8]. Furthermore, ADC is a combined measure of thermally driven molecular movement of water i.e. diffusion and microcirculation of blood in capillaries i.e. perfusion [42] as demonstrated by intravoxel incoherent motion (IVIM) model. Indeed, the IVIM model has been used to study various cancer types [31, 32]. The signal from blood flow is rapidly attenuated at low b values (b < 100–150 s/mm^2^), whereas higher b values are required to suppress the perfusion contribution [43, 44].

One of the most sensitive measures to monitor the tumor progression, in our animal model, was MTR (%). Previous studies have demonstrated that the transgenic *KPC* mouse model has the highest degree of fibrotic tissue accumulation as compared to other mouse models evaluated [45]. Furthermore, studies have demonstrated that MTR values are sensitive to fibrotic tissue deposition and have been used to study liver fibrosis and PDA [17, 18, 34]. Additionally, a study by Farr et al. has demonstrated a significant correlation between MTR and fibrotic tissue deposition in the *KPC* mouse model [18]. Furthermore, using a xenograft mouse model, Li et al. have demonstrated a significant correlation between MTR and fibrotic tissue deposition in different cell lines when the tumor size was close to 10 mm [17]. Similarly, in our study, we have demonstrated a significant correlation between MTR and tumor volume especially when the tumor volume exceeded 250 mm^3^. Whereas when the tumor volume was less than 250 mm^3^, we did not find any correlation between MTR and tumor volume. Additionally, we found that there was a moderate correlation between an increase in tumor volume and fibrotic tissue deposition and a strong correlation between MTR and accumulation of fibrotic tissue suggesting that with an increase in tumor volume there is increase in fibrotic tissue deposition and that MTR changes are sensitive to accumulation of fibrotic tissue in the tumor. Therefore, MTR may be a valuable measure in evaluation of novel treatments and more importantly, in determining the stage of cancer and planning the treatment regimen accordingly.

Finally, APT imaging has been used to measure the concentration of endogenous mobile proteins and peptides which are increased in high-grade brain tumors compared with low-grade tumors [46]. We, however, did not find any significant increase in APT signal intensity when comparing tumor in small and large tumor groups. Surprisingly, in the smaller tumor group, we found a significant decrease in the amide proton group with an increase in tumor volume suggesting that these protons have an important role during early stages of tumor development. Once a threshold value is reached, the concentration of amide protons remained unchanged, suggesting a role of other chemical constituents in higher tumor volumes. Indeed, studies of PDA have suggested that there is increased accumulation of hyaluronic acid (HA) rather than an increase in tumor cellularity, which may be one of the major factors leading to an increase in tumor volume [47, 48].

The multi-parametric approach has been utilized before to characterize various tumors and therapeutic effects [18, 49]. To the best of our knowledge this is the first study to characterize the *KPC* mouse model using a host of MR measurements. Our study has a few limitations that need to be acknowledged. First, due to the dynamic nature of the tumor microenvironment, a host of pathological processes such as necrosis and fibrotic tissue accumulation occur simultaneously with tumor development and future studies need to be conducted in *KPC* mice at various stages of tumor development. Additionally, MR results need to be correlated with histological measurements in detail. In this study, we did not validate the MR measures with the pathological events at different stages of tumor development. Second, we did not quantify HA accumulation, which correlates with high tumor interstitial fluid pressure (IFP) which in turn collapses the surrounding capillaries. Future studies looking at these features and corroboration with histological measurements are warranted.

## Conclusions

We believe that quantitative mp-MRI has a potential role in the monitoring of disease progression and therapeutic evaluation of tumors. In our study, we have demonstrated that MR parameters such as T_1_ and APT are sensitive to changes in the tumor microenvironment during the early stages of tumor development whereas parameters such as T_2_, MTR and ADC are sensitive to pathology during the later stages of tumor development. Additionally, our multi-parametric data suggests that changes in advanced MR techniques such as MTR, ADC and APT imaging have the potential to be used in both preclinical and eventually in clinical models to document the underlying pathophysiological processes and thereby initiating tumor targeting therapy.

## Additional file

Additional file 1: **Figure S1.** Time course multiparametric MR images with colorized MR maps overlaid on T_2_ weighted anatomic images obtained at weeks 1, 2, 3 and 4. (TIFF 1522 kb)

## Abbreviations

APT: amide proton transfer
AUC: area under curve
CEST: chemical exchange saturation transfer
DWI: diffusion weighted imaging
MTR: magnetization transfer ratio
PDA: pancreatic ductal adenocarcinoma
