# Supplementary figures and images for: Evaluation of pancreatic tumor development in KPC mice using multi-parametric MRI
Source: Cancer Imaging. 2018 Nov 8;18:41. doi: 10.1186/s40644-018-0172-6 (PMC6225661; doi:10.1186/s40644-018-0172-6)

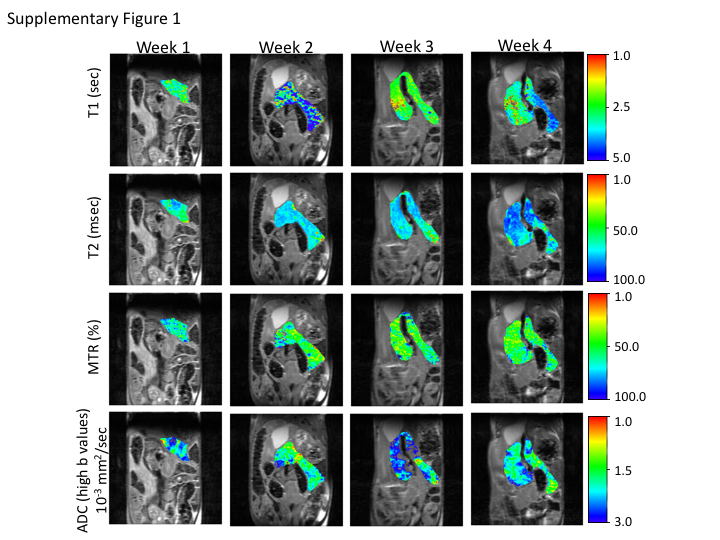

Supplement: Supplementary file 1 — Figure S1. Time course multiparametric MR images with colorized MR maps overlaid on T2 weighted anatomic images obtained at weeks 1, 2, 3 and 4. (TIFF 1522 kb) [file 40644_2018_172_MOESM1_ESM.tiff]
